# 2D µ-Particle Image Velocimetry and Computational Fluid Dynamics Study Within a 3D Porous Scaffold

**DOI:** 10.1007/s10439-016-1772-6

**Published:** 2016-12-12

**Authors:** A. Campos Marin, T. Grossi, E. Bianchi, G. Dubini, D. Lacroix

**Affiliations:** 1 Insigneo Institute for in silico Medicine, Department of Mechanical Engineering, University of Sheffield, Pam Liversidge Building, Mappin Street, Sheffield, S1 3JD UK; 2 Laboratory of Biological Structure Mechanics, Politecnico di Milano, Milan, Italy

**Keywords:** Microfluidics, Tissue engineering scaffolds, Imaging, Computational model, Mass transport properties

## Abstract

Transport properties of 3D scaffolds under fluid flow are critical for tissue development. Computational fluid dynamics (CFD) models can resolve 3D flows and nutrient concentrations in bioreactors at the scaffold-pore scale with high resolution. However, CFD models can be formulated based on assumptions and simplifications. μ-Particle image velocimetry (PIV) measurements should be performed to improve the reliability and predictive power of such models. Nevertheless, measuring fluid flow velocities within 3D scaffolds is challenging. The aim of this study was to develop a μPIV approach to allow the extraction of velocity fields from a 3D additive manufacturing scaffold using a conventional 2D μPIV system. The μ-computed tomography scaffold geometry was included in a CFD model where perfusion conditions were simulated. Good agreement was found between velocity profiles from measurements and computational results. Maximum velocities were found at the centre of the pore using both techniques with a difference of 12% which was expected according to the accuracy of the μPIV system. However, significant differences in terms of velocity magnitude were found near scaffold substrate due to scaffold brightness which affected the μPIV measurements. As a result, the limitations of the μPIV system only permits a partial validation of the CFD model. Nevertheless, the combination of both techniques allowed a detailed description of velocity maps within a 3D scaffold which is crucial to determine the optimal cell and nutrient transport properties.

## Introduction

The flow environment inside 3D porous scaffolds modulates key aspects of in vitro tissue engineering (TE) such as the transport of cells towards the scaffold substrate during cell seeding^13^,^21^,^31^ or the spatial distribution of nutrients and oxygen which is related to cell growth and viability.^10^,^12^,^20^ Moreover, the fluid shear stress exerted on cells affects cell response.^2^,^3^,^27^ Therefore, the acquisition of the spatial fluid flow conditions inside scaffolds is essential to understand the fluid-induced cell behaviour and control tissue development. Computational Fluid Dynamics (CFD) simulations can calculate 3D flow fields with high resolution permitting researchers to optimise hydrodynamic bioreactors and scaffold design for tissue regeneration therapies while avoiding trial and error experiments. For instance, Melchels et al.^19^ investigated the effect of scaffold pore size on the shear rate and its effect on cell adhesion. Zermatten et al.^36^ compared the flow field inside a scaffold with regular microstructure and another with an irregular pore network showing that in irregular scaffold networks the streamlines follow preferable channels so no regular distribution of cells or nutrients can be reached. Furthermore, scaffold location inside the bioreactor and flow rate are also key parameters in perfusion systems as demonstrated by Papantoniou et al.^24^ The geometry of the chamber can also modify the flow profile inside the scaffold as shown by Hidalgo-Bastida et al.^9^ where a circular and a rectangular bioreactor-system were compared.

Despite the potential of CFD simulations to optimise TE processes inside dynamic bioreactors, computational models can be formulated based on assumptions. Thus, experimental measurements should be carried out to verify the reliability of such CFD models. µ-Particle Image Velocimetry (µPIV) has been widely used to measure local fluid velocities and derived properties in microflows. Conventional µPIV consists of illuminating the fluid flow that contains tracer particles with a pulsed laser and capturing the reflected light with a high speed camera in double frame images under a specific time step. Then, velocity vector maps are generated by applying PIV cross-correlation methods.^29^ CFD models that are formulated and validated using µPIV methods hold the potential to substitute physical experiments becoming a virtual unlimited source of trials. Unfortunately, little has been done to characterise the fluid flow inside scaffolds using µPIV methods since they need optical access to the region of interest and most of TE scaffolds are made of non-transparent materials. Despite this barrier, different approaches have been followed to extract representative fluid flow data from 3D scaffolds.

Song et al.^30^ used µPIV to assess the ability of CFD to predict the local fluid-induced microenvironment around cells within scaffolds. As classical µPIV only permits 2D measurements, they calculated shear stress on transverse and axial scaffold sections which exemplify the main 3D architectural features of the scaffold while allowing optical access for the µPIV. Nevertheless, 3D flow environments are found inside scaffolds. It was shown in the literature that the shear stress values to which cells respond can differ significantly from 2D to 3D environments.^16^,^33^ For this reason, De Boodt et al.^6^ claimed that µPIV experiments cannot be performed on 2D substrates and they introduced the 2D+ concept by using a patterned substrate based on a unit cell of a 3D AM (Additive Manufacturing) scaffold where in-plane velocities could be measured. Moreover, De Boodt et al. used µPIV measurements not only for CFD validation but also as feedback to improve the definition of the CFD model; they found significant differences between µPIV and CFD results mainly due to the use of an idealized CAD geometry in the computational model instead of considering the actual scaffold geometry. A similar strategy was followed by Provin et al.^26^ investigating a microstructure compounded by a pillar bundle in a parallel plate chamber to optimise scaffold design and achieve a trade-off between high supply of medium for cells and low shear stress values.

The aim of this study was to resolve the flow field inside a 3D AM scaffold performing µPIV experiments without utilising adapted architectures that are normally used to overcome the limitations of conventional µPIV systems. The approach of this study allows the measurement of velocity fields at the scaffold pore level in a 3D environment using a 2D µPIV system. It is noteworthy that the study focuses on determining scaffold transport properties for cell seeding and culture under fluid flow. Thus, a perfusion system was selected in this study since it seems the most preferable solution to enhance the transport of cells, oxygen and nutrients and waste removal while exposing cells to shear stress inside scaffolds.^17^ The experimental conditions were modelled computationally including the μ-Computed Tomography (CT) geometry of the 3D scaffold. The µPIV measurements were compared to CFD results to evaluate the reliability of the CFD model to describe velocity maps within a 3D pore.

## Materials and Methods

### Experimental Methods

**Figure 1:**
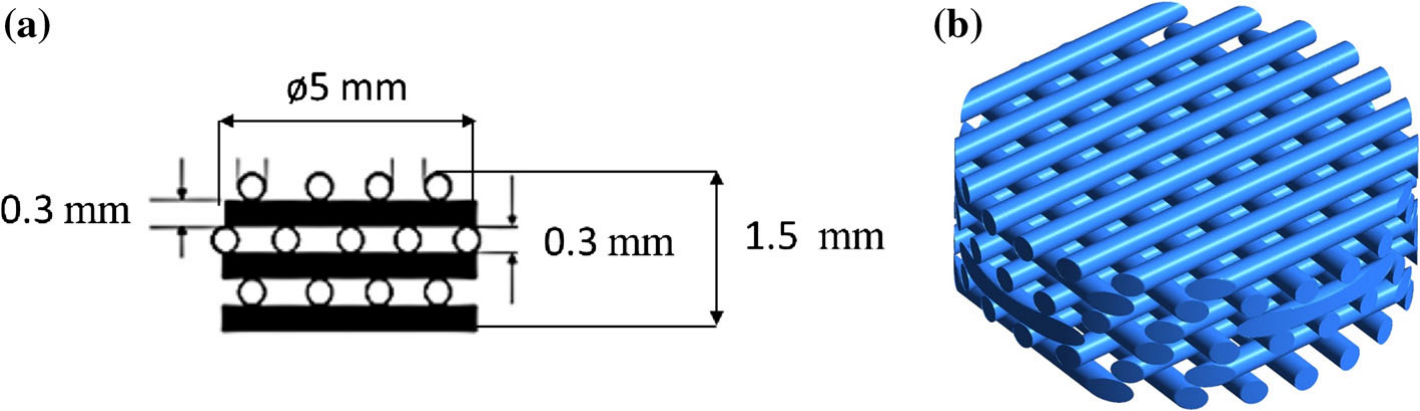
(a) Scaffold design specifications. (b) 3D CAD model of the scaffold.

**Figure 2:**
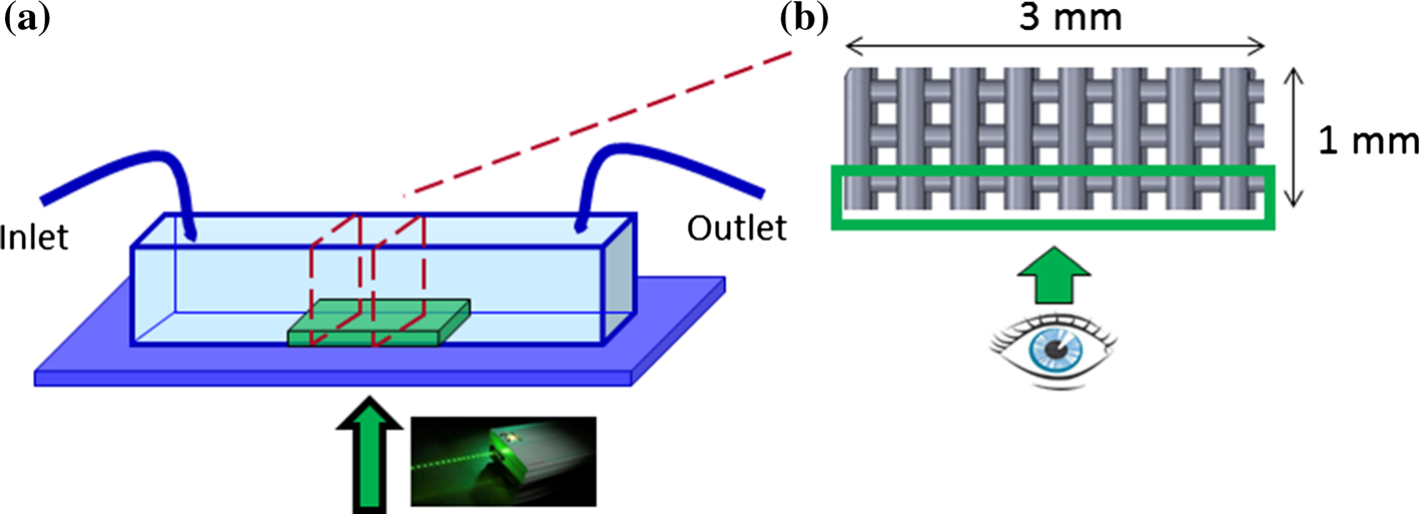
(a) Microfluidic chamber made of PDMS mounted on the microscope stage. The trimmed scaffold (b) was placed inside the rectangular channel to allow optical access to the µPIV system.

A commercial Polycaprolactone scaffold from 3D Biotek (New Jersey, USA) was selected for this study (see Fig. 1). The cylindrical scaffold was trimmed and located inside a micro-channel with rectangular profile to allow optical access to the µPIV system inside the scaffold and therefore quantify the flow field near the scaffold fibres. The depth of field of the µPIV system permitted to focus the working plane within the first layer of pores that consisted of a series of vertical and horizontal fibres arranged in 3D (see Fig. 2). The micro-chamber was made of Polydimethylsiloxane (PDMS) with the following dimensions; 3 × 1 × 40 mm^3^. The chamber was mounted on a surface glass by plasma-activated bounding. A machined mould made of Poly(methyl methacrylate) was used to build the chambers thereby ensuring reproducibility among experimental trials.

#### Microfluidic System

One micrometer diameter polystyrene fluorescent tracer particles (orange, 540/560 nm) were diluted in deionized water with a concentration of 2 × 10^8^ beads/mL. A syringe pump (Harvard Apparatus PhD 2000) was connected at the inlet of the chamber to infuse the working fluid with a constant flow rate of 18 μL/min corresponding to 0.1 mm/s at the scaffold entrance. The outlet of the chamber was connected to a tube that drove the fluid towards a reservoir.

#### μPIV Experimental Procedure

The microfluidic chamber was placed on top of an inverted Olympus IX71 microscope stage with ×10 optics magnification. The µPIV system (TSI Incorporated, Minneapolis, USA) included a synchronised laser (Nd:YAG 532 nm) which was used to excite the tracer particles at two time points with an interval of 10,000 µs. The emitted light from the particles was recorded by a camera (Power View 4 M, 2048 × 2048 pixels) in double frame images. The time interval was selected to obtain particles displacement of 6–12 pixels from frame to frame to facilitate further post-processing. 50 double frame images were combined to reach at least five particles per interrogation region in order to calculate the velocity field accurately. The field of view was 0.94 × 0.94 mm^2^ and the regions investigated were in the vicinity of the fibres. Noise background was subtracted from raw images and the resulting images were processed with a Gaussian filter. Velocity vector maps were calculated by using 25% overlap with the Recursive Gaussian algorithm of Insight 3G (TSI Incorporated, Minneapolis, USA). The calculated velocity fields were analysed in Tecplot (Tecplot, Inc., Bellevue, WA, USA).

However, due to the optics and the size of the tracer particles, out-of-focus particles within a specific depth could contribute to the velocity correlations algorithm. This depth is commonly known as depth of correlation (DOC) and for this setup it is ~25 µm which was calculated using Eq. 1 proposed by Olsen and Adrian^22^:

1 $${\text{DOC}} = 2\left[ {\frac{1 - \sqrt \varepsilon }{\sqrt \varepsilon }\left( {f^{{\#}^{2}} {d^{2}\!\!}_{\text{p}} + \frac{{5.95(M + 1)^{2} \lambda^{2} f^{{\#}^{4}} }}{{M^{2} }}} \right)} \right]^{{{\raise0.7ex\hbox{$1$} \!\mathord{\left/ {\vphantom {1 2}}\right.\kern-0pt} \!\lower0.7ex\hbox{$2$}}}}$$

where, magnification, *M* = 10; wavelength of the light emitted by the particles, *λ* = 0.532 µm; diameter of the particles, *d*_p_ = 1 µm; threshold value to determine the contribution of a particle to the measured velocity, *ε* = 0.01 and focal number, *f* ^*#*^ is calculated by Ref. 18:

2 $$f^{\# } = \frac{1}{2}\left[ {\left( {\frac{{n_{\text{o}} }}{\text{NA}}} \right)^{2} - 1} \right]^{1/2}$$

where *n*_o_, refractive index = 1 and numerical aperture, NA = 0.3.

Since the depth of the pore was around 300 µm, the effect of calculated DOC on the velocity measurements was considered negligible.

A preliminary study in a simple scenario was carried out to determine the accuracy of the system by comparing the µPIV measured velocities of a laminar flow inside the rectangular channel without scaffold with the analytical and CFD solutions.

### Computational Methods

#### CFD µCT-Based Simulations

**Figure 3:**
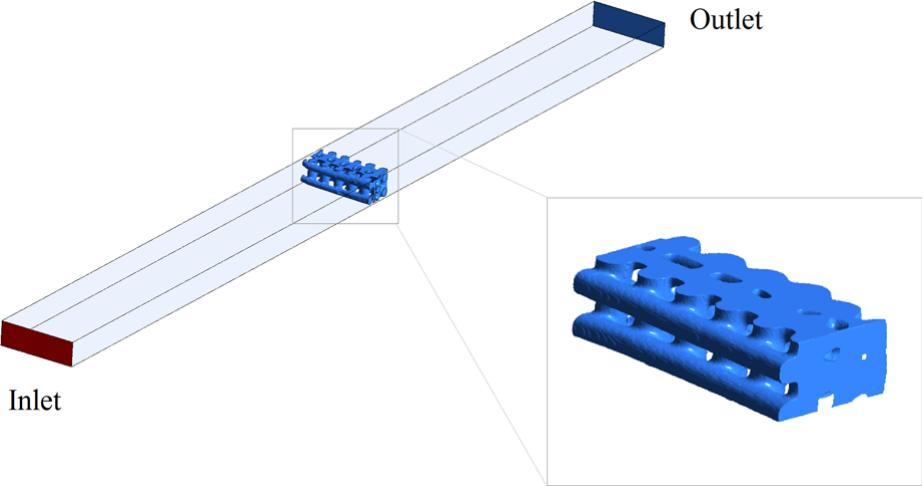
Geometrical boundary conditions of the CFD model (left) and 3D digital reconstruction of the trimmed scaffold using µCT data (right).

The trimmed scaffold was scanned using μCT (Skyscan1172, Materialise, Belgium) at 59 kV voltage and 149 µA beam current with 7 × 7 × 7 μm^3^ of voxel size. The µCT images data were reconstructed with Simpleware (Simpleware Ldt, Exeter, UK). Then, a surface triangular mesh was generated to represent the µCT-based scaffold geometry. The STL mesh of the trimmed scaffold was imported into ICEM (ANSYS Inc., Canonsburg, PA, USA) and located inside a CAD-based rectangular channel following the specifications of the experimental microfluidic chamber (see Fig. 3). The fluid domain was meshed with tetrahedral elements using the robust octree algorithm. Mesh sensitivity analysis was carried out and as a result, around 4 million elements represented the fluid domain. The fluid mesh was modelled in Fluent 15.0 (ANSYS Inc., Canonsburg, PA, USA) as an incompressible Newtonian fluid with dynamic viscosity of 0.001 Pa s and density of 1000 kg/m^3^ representing the deionized water from the experiments. The fluid flow was described by the 3D Navier–Stokes equation. A steady state laminar flow was simulated with a mass flow rate of 18 μL/min at the inlet which corresponds to an average velocity of 0.1 mm/s at the scaffold entrance. Zero pressure at the outlet and no-slip wall conditions were adopted. Simulations were carried out on the Iceberg high performance computing facilities centrally provided by the University of Sheffield using 8 cores in a 2*8-core Intel E5-2670 machine with 256 GB of memory.

## Results

### Accuracy of the µPIV System (Flow in a Rectangular Duct)

The maximum velocity of a laminar flow inside a rectangular duct is not exactly twice the average velocity as found in circular pipes. For this reason, Martineli and Viktorov^15^ presented the formula seen in Eq. 3, where *h* and *w* are the duct height and width respectively, to calculate the ratio of maximum velocity to average velocity as a function of the channel aspect ratio (*h/w*) for incompressible flows:

3 $$v^{*} = \frac{{v_{\hbox{max} } }}{{v_{\text{average}} }} = - 0.56\left( {\frac{h}{w}} \right)^{2} + 1.15\left( {\frac{h}{w}} \right) + 1.5$$

**Figure 4:**
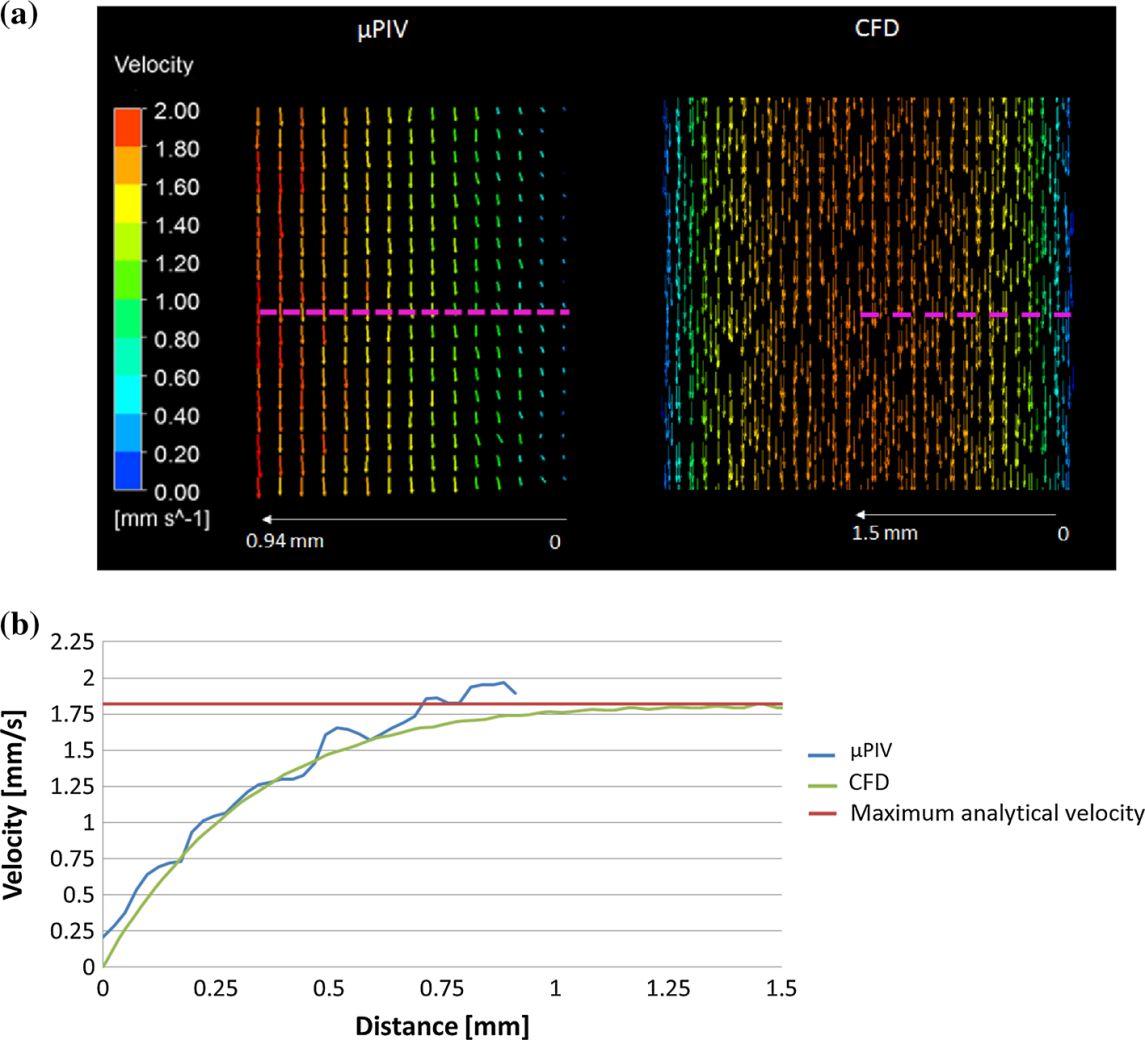
(a) Velocity vectors from a plane located in the middle of the rectangular channel calculated using µPIV (left) and CFD (right) methods. The pink dotted lines show from where the velocity values were extracted to compare both techniques quantitatively. (b) The blue line and green lines represent the velocity values extracted from the profiles shown in (a) for the µPIV and CFD tools, respectively. The red line is the maximum fluid velocity calculated analytically that can be reached inside the rectangular channel.

The average inlet velocity is 1 mm/s for this test and the channel aspect ratio is 1/3; therefore, the expected maximum velocity at 1.5 mm distance from the lateral channel wall, corresponding to the centre of the channel, should be 1.82 mm/s. The CFD model calculates a maximum velocity of 1.83 mm/s at the centre of the channel so it agrees well with the analytical value calculated using the formula in Eq. 3. In the case of µPIV, the velocity extracted from the pink line in Fig. 4a reaches 1.89 mm/s at 0.9 mm distance from the channel wall, as seen in Fig. 4b, whereas the CFD value at that location is 1.73 mm/s. Assuming that the CFD can predict with accuracy the fluid velocity profiles, the expected error from the µPIV to calculate fluid velocities is ~10% for the specific experimental scenario implemented in this study with a tendency to overestimate the velocity values.

### Local Fluid Velocities Inside the Scaffold Measured with µPIV

**Figure 5:**
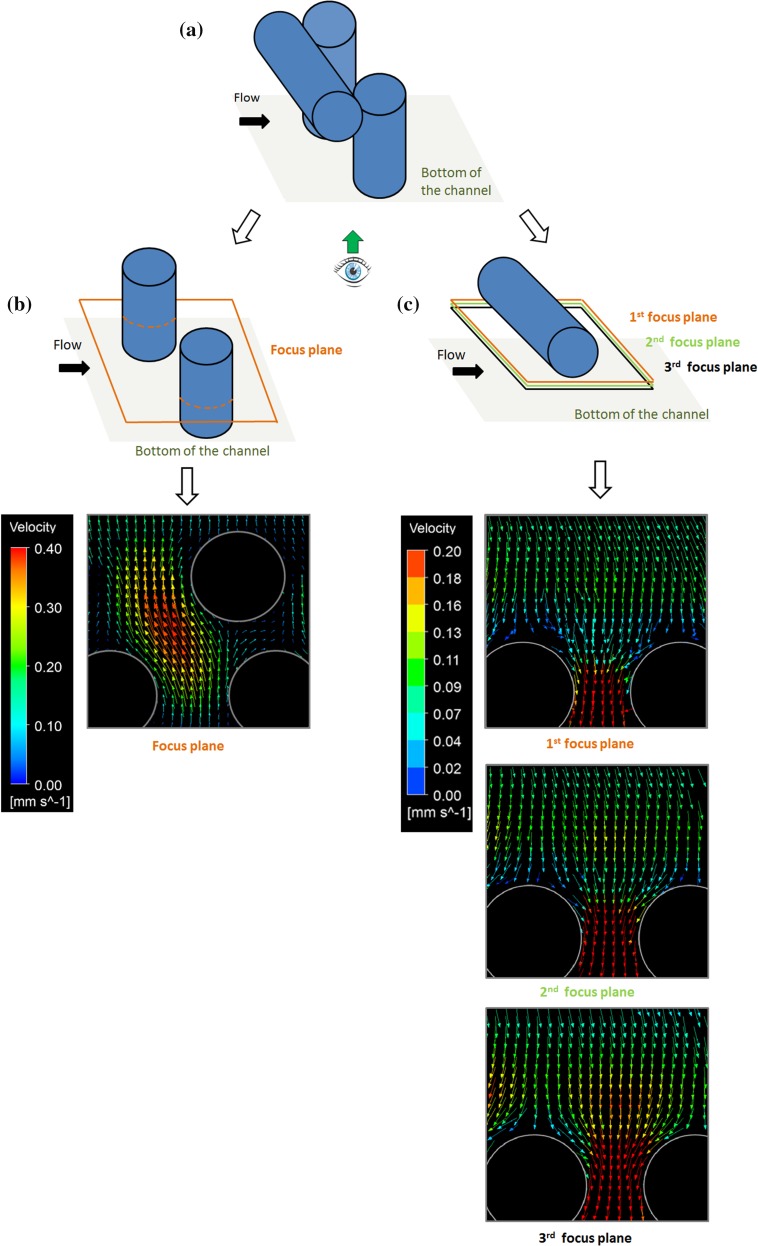
(a) Representation of scaffold pore where the flow field is analysed. (b) Velocity vectors between vertical fibres calculated with µPIV. (c) Velocity vectors from the 1st (a), 2nd (b) and 3rd (c) planes underneath the horizontal fibres calculated with µPIV within the scaffold pore.

Two regions of interest were considered to characterize the fluid flow inside the scaffold pores, both parallel to the flat glass surface. The fluid flow passing between the vertical fibres was observed, as well as the fluid flow underneath the horizontal fibre, as shown in Fig. 5a. It is worth noting that μPIV measured velocities can represent the in-plane components, only.

The velocity of the fluid flow passing between the vertical fibres (see Fig. 5b) shows maximum values at the centre of the pore and it decreases towards the wall of the fibres. On the other hand, three different working planes were set to investigate the velocity gradients when moving down away from the horizontal fibre (see Fig. 5c). The measured velocities increase with the distance of the focus plane from the fibre. When observing the area inside the pink box shown in Fig. 5c, the no-slip wall effect on the fluid velocities is reduced when moving away from the horizontal fibre from the first to the third focus plane. Moreover, the velocity maps are closer to the expected continuity as the fluid velocity has to increase when it is forced to flow through a smaller area.

### Comparison CFD-μPIV

**Figure 6:**
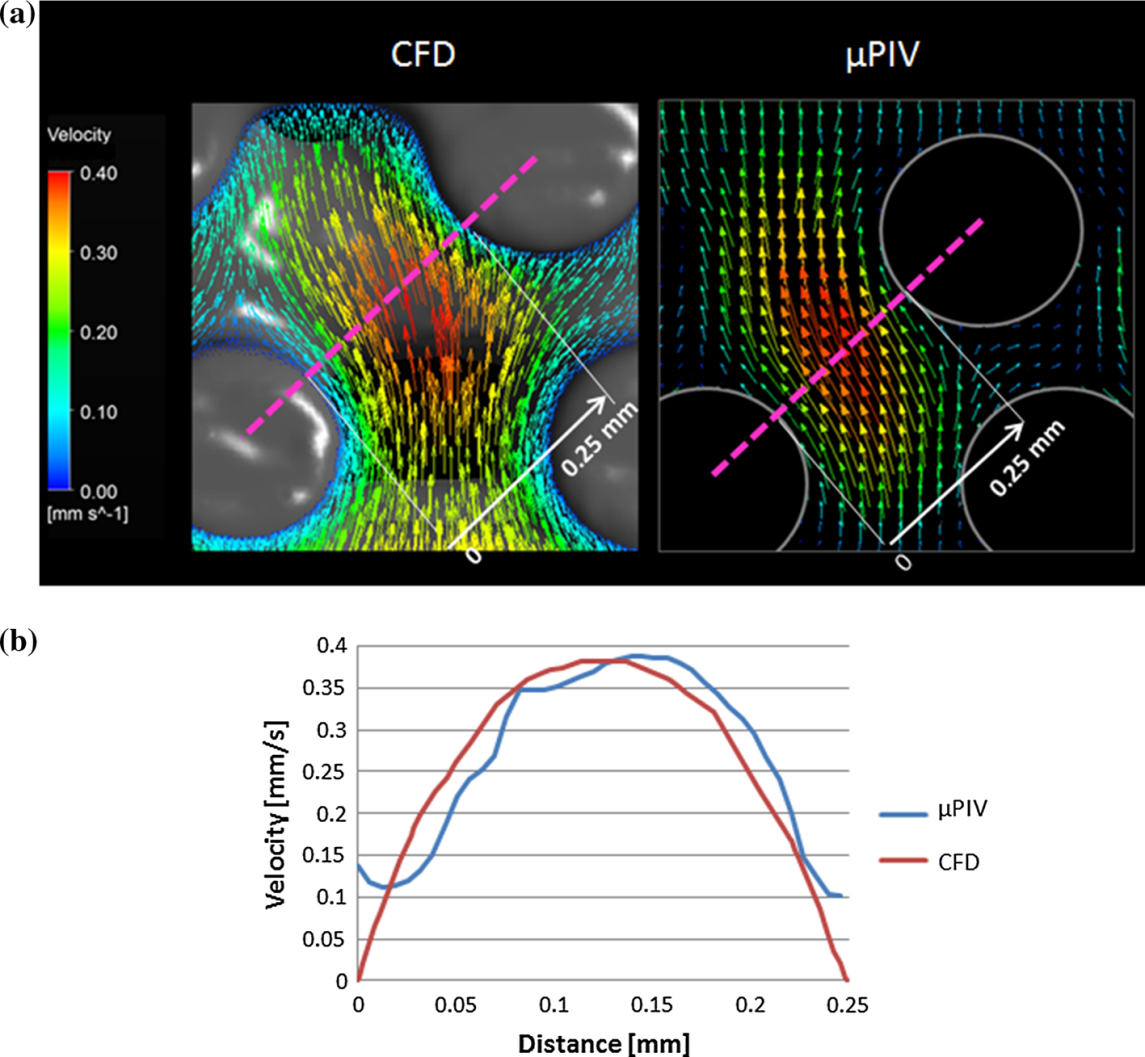
(a) Velocity vectors from a plane inside a pore between the vertical fibres calculated using µPIV (right) and CFD (left) methods. The pink dotted line shows where the velocity values were extracted to compare both techniques quantitatively. (b) The blue and red lines represent the velocity values extracted from the profiles shown in (a) for the µPIV and CFD, respectively.

**Figure 7:**
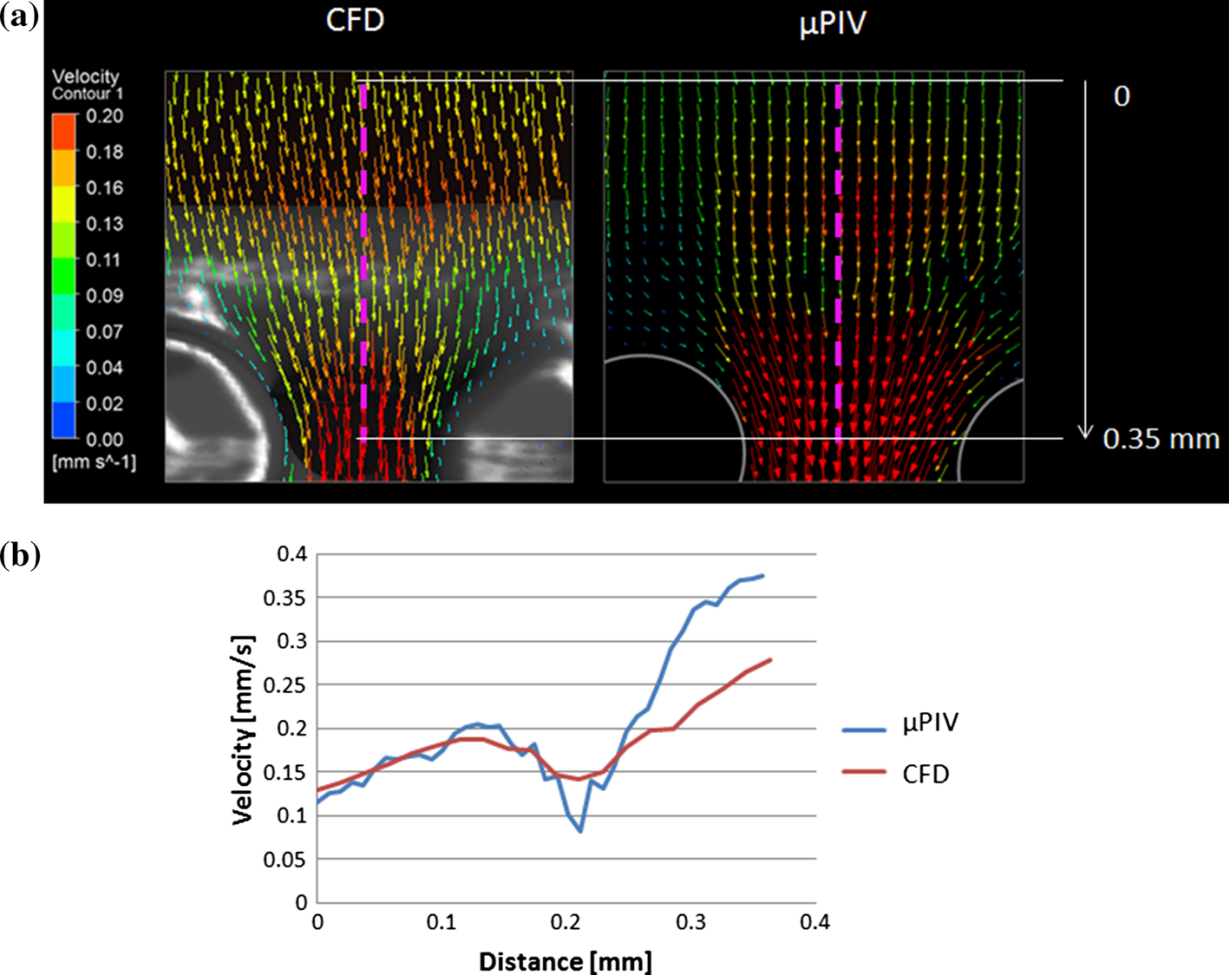
(a) Velocity vectors from the second focus plane underneath the horizontal fibre calculated using µPIV (right) and CFD (left) methods. The pink dotted lines shows where the velocity values were extracted to compare both techniques quantitatively. (b) The blue and red lines represent the velocity values extracted from the profiles shown in (a) for the µPIV and CFD, respectively.

**Figure 8:**
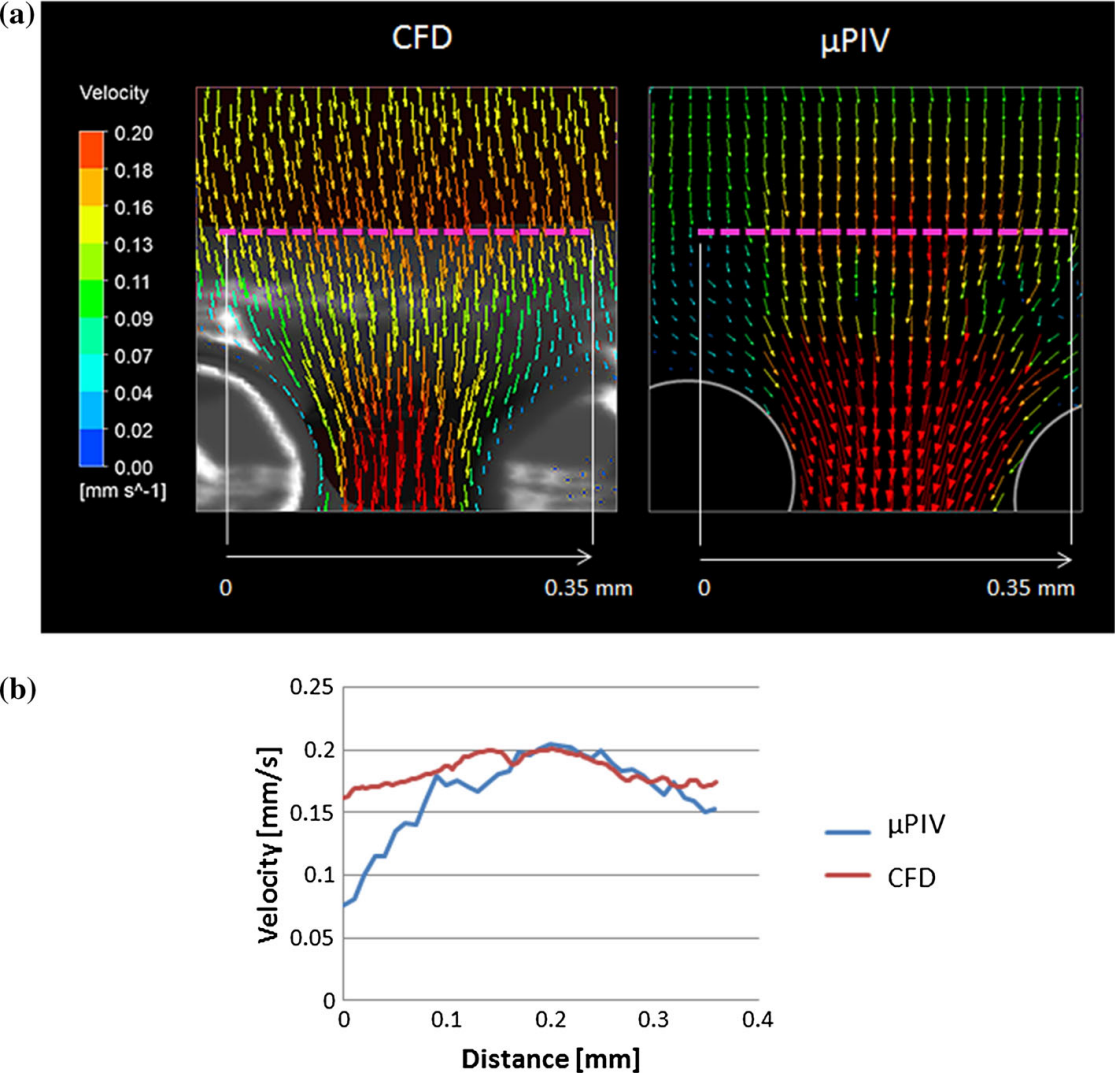
(a) Velocity vectors from the second focus plane underneath the horizontal fibre calculated using µPIV (right) and CFD (left) methods. The pink dotted lines shows where the velocity values were extracted to compare both techniques quantitatively. (b) The blue and red lines represent the velocity values extracted from the profiles shown in (a) for the µPIV and CFD, respectively.

The CFD results agree well with the velocity profiles calculated using the μPIV system in the scaffold regions seen in Figs. 6, 7, and 8. First, both approaches show that peak velocities are found at the centre of the pore defined by the vertical fibres as observed in Fig. 6 and that they decrease when approaching the wall fibres. The good agreement between the experimental and computational approaches is not only qualitative but also in terms of velocity magnitude, which is due to the fact that the fluid velocity component in the transversal direction is almost zero so fluid velocity vectors mostly fall in the focus plane. There is only a maximum difference of 12% in velocity magnitude inside the pore. However, when reaching the fibres walls, the µPIV velocities are non-zero, in contrast with the CFD results where no-slip condition was applied (see Fig. 6b).

The CFD and µPIV results show the same trend underneath the horizontal fibre as observed in Fig. 7; the fluid velocity starts increasing in the region where the flow encounters the horizontal fibre on its path and then decreases just before entering in the pore formed by the vertical fibres. The region where the velocity drops is the closest part of the focus plane to the horizontal fibre; thus, the no-slip wall effect reduces the fluid velocity. In theory, the velocity of the incompressible fluid should increase as travelling towards the pore formed by the two vertical fibres where the area through which the fluid flows is smaller. Therefore, the velocity should increase to obey continuity. In terms of velocity magnitude, the agreement between both techniques becomes poorer as the fluid enters the pore with up to 70% difference (see Fig. 7b).

In addition, the CFD and the µPIV results showed the same peak velocity value at the central position of the horizontal fibre which is aligned to the centre of the pore formed by the two vertical fibres as shown in Fig. 8. Moreover, in both methods the velocities decrease as moving away from the centre of the horizontal fibre, although velocity values can differ up to 46% (see Fig. 8b).

## Discussion

The velocity profiles inside a non-transparent 3D scaffold were resolved by μPIV methods. The depth of field of the μPIV system permitted to focus the working plane within the first layer of pores of the trimmed scaffold that consisted of a series of vertical and horizontal fibres arranged in 3D. Despite the 3D configuration of the observed pores and the expected 3D motion of the tracer particles, valuable data could be extracted using a conventional µPIV system. The fluid flow was measured between the two vertical fibres within a focus plane that was parallel and sufficiently close to the flat surface at the bottom of the channel. Therefore, fluid velocity vectors mainly had in-plane components. Similar results occurred when analysing the fluid flow close to the horizontal fibre; velocity vectors tend to align to the fibre surface and thereby to the working place. Thus, the conventional μPIV system used in this study that only can measure 2D particles displacements served to analyse the fluid flow velocities inside the 3D pores of the selected scaffold. The main velocity profiles inside the scaffold were described; the fluid velocities between the vertical fibres are higher at the centre of the pore and the effect of the horizontal fibre on the velocity gradients over the pore depth could be captured.

The experimental μPIV data served to obtain representative fluid velocity data at the pore scale within a 3D AM scaffold. However, as discussed by Campos Marin and Lacroix,^5^ variability in terms of pore velocities can be expected from pore to pore and from scaffold to scaffold due to alterations in scaffold micro-architecture during the fabrication process. Therefore, the μCT-based geometry of the trimmed scaffold was included in the CFD model to be able to analyse the same region using both techniques. As a result, both techniques agreed well in the description of the main velocity behaviour found with the μPIV system.

The CFD simulations could predict the analytical solution for the maximum velocity of a fully developed fluid flow inside a channel rectangular profile. Considering that the CFD resolved the fluid flow accurately in that case, the μPIV system had a maximum error of 10%. Therefore, when measuring fluid velocities inside the scaffold some differences between both techniques can be expected due to the accuracy of the μPIV plus the fact that the CFD model can have some simplifications of reality. The quantitative comparison of the fluid velocities between the vertical fibres shows a maximum error of 12% which is acceptable regarding the error of the μPIV system found in the square channel. However, it is observed that close to the walls μPIV-calculated velocities are non-zero on the contrary as assumed in the CFD model. This could be explained by the lack of resolution of the μPIV system being unable to capture the no-slip very close to the walls or the noise due to scaffold brightness that contributed to the calculation of the velocity maps. For the analysis in the vicinity of the horizontal fibre, the horizontal fibre induces parallel fluid velocities to its surface and in-plane velocity vectors in the focus plane. Therefore, good agreement was found close the fibre. However, for the rest of the fluid velocities calculated in the same focus plane, a difference of up to 70% was found between CFD and μPIV. This is due to the fact that out-of-plane fluid velocity vectors are expected in some regions of the focus plane due to the 3D geometry of the scaffold.

The measurement of 3D velocities could be addressed by using calibration methods such as the one presented by Winer et al.^34^ where the particle z-position is correlated to its apparent diameter. Another option to measure 3D fluid velocities is stereoscopic PIV^7^ that uses more than one capturing system in a stereoscopic arrangement. However, this leads to optical access constraints when investigating 3D scaffolds. Nevertheless, this method was successfully applied to calculate the fluid dynamics around a 3D scaffold in a stirring bioreactor where the effect of the bioreactor rotation rate was related to mixing properties.^8^ Other promising methods such as the defocusing method can also detect 3D particles displacements, although to the authors’ knowledge, it has not been applied yet to investigate TE scaffolds. It consists of an aperture located on the objective lens that contains three pinholes forming an equilateral triangle. The light from the particle passes the aperture and then reaches three different positions at the image plane being able to determine the particle position with respect to the focus plane by measuring the distance between the projected triangle vertices.^35^ It is noteworthy to mention that nuclear magnetic resonance can measure 3D flows inside opaque materials as shown by Mack et al.^14^ who captured the local hydrodynamics inside a 3D porous scaffolds made of PCL. However, 1 mm^3^ of spatial resolution was not enough to calculate the local mechanical stimuli at the pore level.

On the other hand, CFD simulations may have some limitations to represent the experimental conditions. For instance, the realistic position of the trimmed scaffold inside the channel is unknown and cannot be incorporated in the CFD model. Empty spaces between the scaffold and the channel walls or the scaffold orientation with respect to the walls and the flow direction could significantly alter the local fluid dynamics. This also could explain some of the disagreements found in terms of velocity magnitude. Furthermore, the selection of the exact μPIV focus plane in the CFD is critical for the adequate comparison of both methods. Moreover, the wall boundaries in the CFD model may not capture the real roughness of the scaffold or channel surfaces which can alter the local fluid flow as shown by Silva et al.^28^ A finer mesh would be necessary to include the surface topography in the CFD model, however; the computational cost was unaffordable at the time. Nevertheless, the reported velocity profiles are expected to be repeated in all scaffold pores although with possibly significant variance in terms of magnitude in the presence of geometrical defects or microstructural variability. The analysis of more pores would be beneficial to obtain statistically significant data but the working fluid stained the scaffold over time thereby being unable to re-use the scaffold in more experiments.

The measurement of the local fluid flow velocities serves to assess the mass transport properties of scaffolds. Fluid flow velocities regulate the spatial distribution of nutrients and oxygen and the removal of cellular wastes which are critical for cell viability. However, in this study no cells were present on the scaffold substrate when resolving the velocity profiles and the presence of cells can alter the local fluid dynamics.^29^ The present study rather investigates the initial fluid flow conditions prior to cell seeding and thereby cell transport properties of the scaffold. The fluid flow has a strong impact on the resulting density and spatial distribution of cells inside the scaffold which are related to final tissue properties.^4^ Based on the results presented herein, more cells are expected to pass by the centre of the pores where fluid velocities are higher thereby less cells will travel next to the fibres substrate. Consequently, the probability of cells to intercept the scaffold and therefore to adhere to it will be low, impacting negatively on the initial conditions for tissue development. However, the effect of fluid flow on cell transport should be investigated. To date, cell motion under fluid flow inside scaffolds during cell seeding has not yet been investigated experimentally. Cells could be tracked during cell seeding along time and space using particle tracking methods.^23^,^32^ The present results from μPIV could relate the velocity profiles with cell motion. Thus, these experimental data could help to understand cell motion in suspension flow for optimization of dynamic seeding systems. In parallel, cell transport could be investigated with CFD by including a discrete phase of micro-particles representing cells to the fluid phase as shown by Adebiyi et al.^1^

It is noteworthy that in this study a steady flow was applied. However, pulsatile flows can be more stimulatory than steady flows for tissue growth as shown by Jaasma and O’Brien.^11^ The characterization of unsteady flows using μPIV remains challenging as pairs of images are captured over time and averaged to calculate instantaneous velocity maps. If the fluid flow changed over time those images could not be averaged, as they would capture different fluid flow phases. To address this issue, Poelma et al.^25^ calculated the mean velocity of each pair of images and based on the mean value a flow phase was assigned. Then, images with similar phase were used to calculate the fluid flow profile at that particular phase so the flow field could be resolved temporally.

The transport properties of TE scaffolds under fluid flow affect tissue development. The characterization of fluid flow fields inside 3D scaffolds is crucial for the optimization of scaffold and bioreactor designs. For the first time, fluid velocities were obtained experimentally from the actual 3D scaffold without building adapted geometries to conventional 2D μPIV systems. Valuable data were extracted with μPIV within a 3D pore and used to validate the μCT-based CFD model. Good agreement was found between both methods. However, some quantitative differences show that μPIV lacks of resolution near the substrate of the fibres due to scaffold brightness. Therefore, μPIV could partly serve as a validation tool for the CFD model. On the other hand, the accurate representation of experimental boundary conditions such as surface roughness or geometry using CFD remains challenging. Nevertheless, the coupling of both methods allowed a detailed description of velocity maps where no cells were present. This could be beneficial to optimise the initial conditions of scaffold cell seeding under fluid flow. However to better understand the role of fluid flow in cells transport, cells should be tracked along time and space with optical systems.
